# The effect of a subject-specific AFO on the muscle activation during gait of a test subject suffering from a hemiparetic anterior muscle insufficiency in the lower leg

**DOI:** 10.1186/1757-1146-5-S1-P6

**Published:** 2012-04-10

**Authors:** V Creylman, L Muraru, H Vertommen, L Peeraer

**Affiliations:** 1 Mobilab, University College Kempen, Belgium; 2 Division of Biomechanics, KULeuven, Belgium; 3 Faculty of Kinesiology and Rehabilitation Sciences, KULeuven, Belgium

## Background

An Ankle Foot Orthosis (AFO) is commonly used in clinical practice to assist gait of patients with different pathologies. The flexibility of the AFO depends on different design characteristics while specific mechanical requirements of the AFO are correlated with patient anatomy and pathology. To this day, the correlation between AFO-design and patient pathology is mainly based on the orthopaedic technician’s experience.

The aim of this study is to investigate the influence of the stiffness of an AFO on the muscle-activation pattern of a subject suffering from an anterior muscle insufficiency of the lower leg using a personalized musculoskeletal model.

## Materials and methods

Test subject was a 40-year old male suffering from a hemiparetic anterior muscle insufficiency of the lower leg. A musculoskeletal model of the lower limbs with 23 degrees of freedom and 92 muscles was scaled in OpenSim to match the test subject’s anthropometric data [1]. Muscle-definitions were adapted to simulate the patients’ pathology.

A subject specific AFO was constructed using the selective laser sintering technique [2]. The actual stiffness of the AFO was determined using finite element analysis [3] and was 258 Nm/rad.

Marker trajectories of an AFO-gait were used to calculate kinematic parameters and muscle-activation during gait using the musculoskeletal model. The AFO was simulated as an angle-dependent torque around the ankle with a neutral angle of 0°. The stiffness of the AFO was varied between 150 and 350 Nm/rad in steps of 25 Nm/rad.

## Results

Results showed alternating muscle-activation in the affected lower leg with increasing AFO-stiffness. No clear correlation could be found between AFO-stiffness and muscle-activation.

## Conclusion

An optimal AFO-stiffness in terms of muscle-activation can be selected for the patient. It must be kept in mind that these results are based on a calculation with a neutral AFO ankle position of 0°, and changing this neutral angle, affects the results.
